# Photocatalytic degradation of single and binary mixture of malachite green and rhodamine B dyes by biochar-capped iron oxide nanocomposites

**DOI:** 10.1007/s11356-025-37025-8

**Published:** 2025-10-15

**Authors:** Peter A. Ajibade, Thandi B. Mbuyazi

**Affiliations:** School of Chemistry and Physics, University of KwaZulu-Natal, Private Bag X01, Scottsville, Pietermaritzburg, 3209 South Africa

**Keywords:** Iron oxides, Nanocomposites, Photocatalytic degradation, Binary dye, Malachite green, Rhodamine B

## Abstract

Synthetic dyes widely used in textile are continuously being discharged into water sources and constitute significant hazard to the environment and human health. This study reports the synthesis, characterization, and photocatalytic performance of iron oxide nanocomposites using biochar carbonized at different temperatures for efficient degradation of malachite green (MG) and rhodamine B (RhB) dyes. *Portulacaria afra* leaves were carbonized at 200℃ to prepare Fe_3_O_4_@BC–**1**, at 400℃ to prepare Fe_3_O_4_@BC–**2**, and at 600℃ to prepare Fe_3_O_4_@BC–**3** iron oxide nanocomposites. Powder X-ray diffraction analysis revealed a cubic Fe_3_O_4_ crystalline phase of iron oxide regardless of the carbonization temperature of the biochar. HRTEM images showed different morphologies with average particle sizes of 11.2–13.3 nm. Energy band gaps of 1.85 eV (Fe_3_O_4_@BC–**1**), 1.79 eV (Fe_3_O_4_@BC–**2**), and 1.97 eV (Fe_3_O_4_@BC–**3**) were obtained from the Tauc plot. The as-prepared iron oxide nanocomposites were used as photocatalysts for the degradation of malachite green (MG), rhodamine B (RhB), and their binary mixture under visible light irradiation. Fe_3_O_4_@BC-**2** exhibited the highest photocatalytic degradation efficiencies of 99.74% for MG and 98.89% for RhB in the binary dye mixture. Optimal degradation of malachite green and rhodamine B was achieved at pH 10 and the iron oxide nanocomposites exhibited good photostability and reusability over five consecutive cycles.

**Supplementary Information:**

The online version contains supplementary material available at 10.1007/s11356-025-37025-8.

## Introduction

Organic dyes are widely used in daily life and are a significant component of industrial wastewater (Lin et al. 2023). Malachite green and rhodamine B are synthetic organic dyes often used in textiles dyeing and as biological indicators, but their toxicological properties have raised environmental and health concerns, especially in wastewater treatment (Cheng et al. 2022). These dyes are potential carcinogens, making their presence in effluents a critical environmental issue (Kapanga et al. 2024). While photocatalysis has been extensively studied as a method to remove harmful pollutants from wastewater, most photocatalysis research has focused on degradation of single dyes as contaminants. However, industrial wastewater often contains mixture of dyes rather than just one dye pigment that require the need to evaluate the effectiveness of potential photocatalysts to simultaneously remove/degrade multiple dyes in the same medium or system (Naresh et al. 2018; Malik et al. 2022). Research on the photocatalytic removal of mixed pollutants is both challenging and limited in literature.

Photocatalytic reactions predominantly occur on the surface of the catalyst (Li et al. 2014). Therefore, modification of the adsorption characteristics is a more practical approach to alter the redox energies of materials as highly selective photocatalysts (Qumar et al. 2022). Pollutants that preferentially adsorb to the catalyst surface tend to react more effectively with active radicals, leading to selective photocatalytic activity. Photocatalytic selectivity can be achieved through functionalization with specific groups, doping and alloying (Lee et al. 2023; Dostanić et al. 2024; Kazim et al. 2024). These advancements can contribute to the development of photocatalysts capable of treating complex wastewater discharged by various industries into natural water bodies.

Carbon-based nanomaterials have emerged as one of the most desirable materials for water treatment due to their unique chemical and physical attributes (Shi et al. 2021; Scaria et al. 2022; Soffian et al. 2022). Activated carbon has a high adsorption capacity, but its practical applications can often be restricted due to its expensive and time-consuming production process (Saleem et al. 2019; Satyam and Patra 2024). There is growing interest in the development of sustainable materials for wastewater treatment. Biochar offers a practical alternative to more expensive activated carbon for the removal of organic contaminants due to its low cost. Biochar is prepared through carbonization of biomass in a vacuum (Srivatsav et al. 2020; Zeghioud et al. 2022). Biochar’s porous structure and large surface area enable it to adsorb a wide range of contaminants, such as heavy metals, organic compounds, and pathogens, and thus offer wide versatility in water treatment and decontamination processes. It can be produced from various biomass sources as a cost-effective option for wastewater treatment (Afshar and Mofatteh 2024).

Biochar’s versatility extends beyond adsorption-based remediation. Extensive studies have explored biochar as a support material for immobilization in environmental remediation and enzyme applications (Palansooriya et al. 2022; Vuong et al. 2025). Modified biochar supports have successfully immobilized bacteria such as *Bacillus *spp., facilitating nutrient release and heavy metal stabilization in soils, which improves soil fertility and contaminant retention (Cheng et al. 2025). Through a combination of physical and chemical mechanisms, biochar effectively immobilizes potentially hazardous elements like heavy metals in contaminated soils (Sachdeva et al. 2023). In addition, biochar derived from sour cherry stones has been used for laccase immobilization, with high efficiency in brilliant green degradation (Wang et al. 2024). Despite these advancements, the scalability, long-term stability, and reusability of immobilized biochar systems under complex environmental conditions remain a challenge (Antanasković et al. 2024; Cho et al. 2024).

Studies have also explored modifications such as a combination of biochar with iron oxide nanoparticles to form nanocomposites, which addresses some of biochar’s constraints and enhances its adsorption capabilities for wastewater treatment (Gao et al. 2021; Farahbakhsh et al. 2022; Amdeha 2023). Magnetized biochar integrates the high adsorption capacity of biochar with the pollutant-removal abilities of iron oxide nanoparticles. These nanocomposites with enhanced surface reactivity increase their adsorption through electrostatic attraction or surface complexation (Ahuja et al. 2022). The magnetic properties of the nanocomposites allow for easy separation of the biochar from water using external magnets, simplifying recovery and reuse by reducing the need for filtration or centrifugation. In addition, iron oxide nanoparticles improve the stability and mechanical strength of biochar, which make the as-prepared composite more resistant under extreme acidic or basic conditions in wastewater (Santhosh et al. 2020; Huang et al. 2022; Sadati and Ayati 2023). They also enable photocatalytic or Fenton-like reactions, facilitating the degradation of organic pollutants into less harmful substances and offer additional treatment mechanism beyond simple adsorption (Wang et al., 2023) .

Research has demonstrated that biochar could serve a key role in promoting the separation of photogenerated electron–hole pairs due to their efficient electron flow pathways (Qian et al. 2022; Ma et al. 2024; Wu et al. 2024). Zhang et al. synthesized Cu_2_O/Ag coated with wood-based biochar composite for Congo red and methyl orange dye removal under visible light (Zhang et al. 2024). Gaber et al. reported spinach stalks biochar/ZnO nanocomposite prepared to degrade 99.34% of bromothymol blue after 120 min (Gaber et al. 2024). Samaraweera et al. tuned the Fe_3_O_4_-modified wood-based biochar composite morphology to remove bromophenol blue (Samaraweera et al. 2023). The composite achieved a removal efficiency of 89.8% after 120 min at pH 5. Biochar requires precise control regarding the incorporation of iron oxide nanoparticles onto its surface. It can be difficult to achieve an equal dispersion while maintaining the pore structure of the biochar (Yameen et al. 2024). Consequently, for optimum results, precise preparation of these nanocomposites is still critical.

In this study, we present the preparation of iron oxide nanoparticles capped with biochar derived from *Portulacaria afra* leaves, an eco-friendly and underexplored biomass known for its high carbon dioxide absorption capacity (Tabassum et al. 2023). The effect of biochar carbonization temperature on the morphological and optical properties of the iron oxide nanocomposites was evaluated. The iron oxide nanocomposites were used for the photocatalytic degradation of malachite green, rhodamine B, and their binary mixture to assess the competitive interactions in a multi-contaminant system under visible light. The effect of irradiation time, pH, and scavengers on the photocatalytic degradation efficiency was evaluated to deduce the degradation mechanism. The photostability and reusability of the as-prepared nanocomposite were also assessed, demonstrating its potential for sustainable and long-term application in wastewater treatment.

## Materials and methods

### Chemicals

Materials used for the synthesis of the magnetic nanocomposites are FeCl_2_·4H_2_O, Fe_2_(SO_4_)_3_·H_2_O, 25% ammonia solution, *Portulacaria afra* biochar, and ethanol. The dyes used for the photocatalytic study are malachite green and rhodamine B. Scavengers used were benzoquinone, isopropyl alcohol, and silver nitrate. The pH was adjusted using HCl and NaOH. Potassium dichromate, mercury(II) sulphate, ferrous ammonium sulphate, ferroin indicator, and sulphuric acid were used for COD analysis. All materials purchased from Sigma-Aldrich were used as received.

### Physical characterization

HRTEM images, lattice fringes, and selected area electron diffraction (SAED) patterns were obtained using a JEOL JEM-2100, and particle sizes were measured with ImageJ 1.53t software. Powder X-ray diffractograms were obtained using a Malvern Panalytical Aeris diffractometer with PIXcel detector and fixed slits with Fe filtered Co-Kα radiation. X-ray fluorescence (XRF) analysis was performed using a Panalytical Axio WDXRF spectrometer. Surface functional groups of the nanoparticles were characterized using a Bruker ALPHA II FTIR spectrometer. The specific surface area was determined using a Micromeritics Tristar II 3020 analyzer with nitrogen adsorption at 77 K. Perkin Elmer lambda 25 UV–Vis spectrophotometer was employed for the qualitative analysis of the magnetic nanoparticles and composite (PerkinElmer, Waltham, MA, USA). Dye degradation experiments were conducted under an OSRAM HQL (MBF-U) 125 W lamp, and the pH levels were monitored with a Metrohm 827 pH meter.

### Preparation of biochar

*Portulacaria afra* leaves were collected at Pietermaritzburg area in South Africa. Thirty grams of dried *Portulacaria afra* leaves was placed in a porcelain crucible and carbonized at 200 °C, 400 ℃, and 600 ℃ in a vacuum tube furnace for 10 min. The resulting biochar was then ground into a powder using a pestle and mortar (Nnadozie and Ajibade 2021).

### Synthesis of biochar-capped iron oxides nanoparticles

Iron oxide nanocomposites were synthesized using the reported co-precipitation method (Ajibade and Nnadozie 2023). Fe_2_(SO_4_)_3_·H_2_O (0.005 mol, 2.0895 g) and FeCl_2_·4H₂O (0.0025 mol, 0.4970 g) were dissolved in 50 mL of distilled water in a 3-neck round-bottom flask. The solution was heated to 90 ℃ under a nitrogen atmosphere, followed by the addition of 15 mL of 25% ammonia solution. The mixture was stirred for 1 h, after which 2 g of biochar was added and stirred for a further 6 h. The biochar-capped nanocomposite was then rinsed with distilled water and ethanol and dried in an oven at 80 ℃ for 8 h. The obtained iron oxide nanocomposites were labelled Fe_3_O_4_@BC–**1**, Fe_3_O_4_@BC–**2**, and Fe_3_O_4_@BC–**3** prepared using biochar carbonized at 200 ℃, 400 ℃ and 600 ℃, respectively.

### Photocatalytic studies of biochar-capped iron oxide nanoparticles

**Fig. 1:**
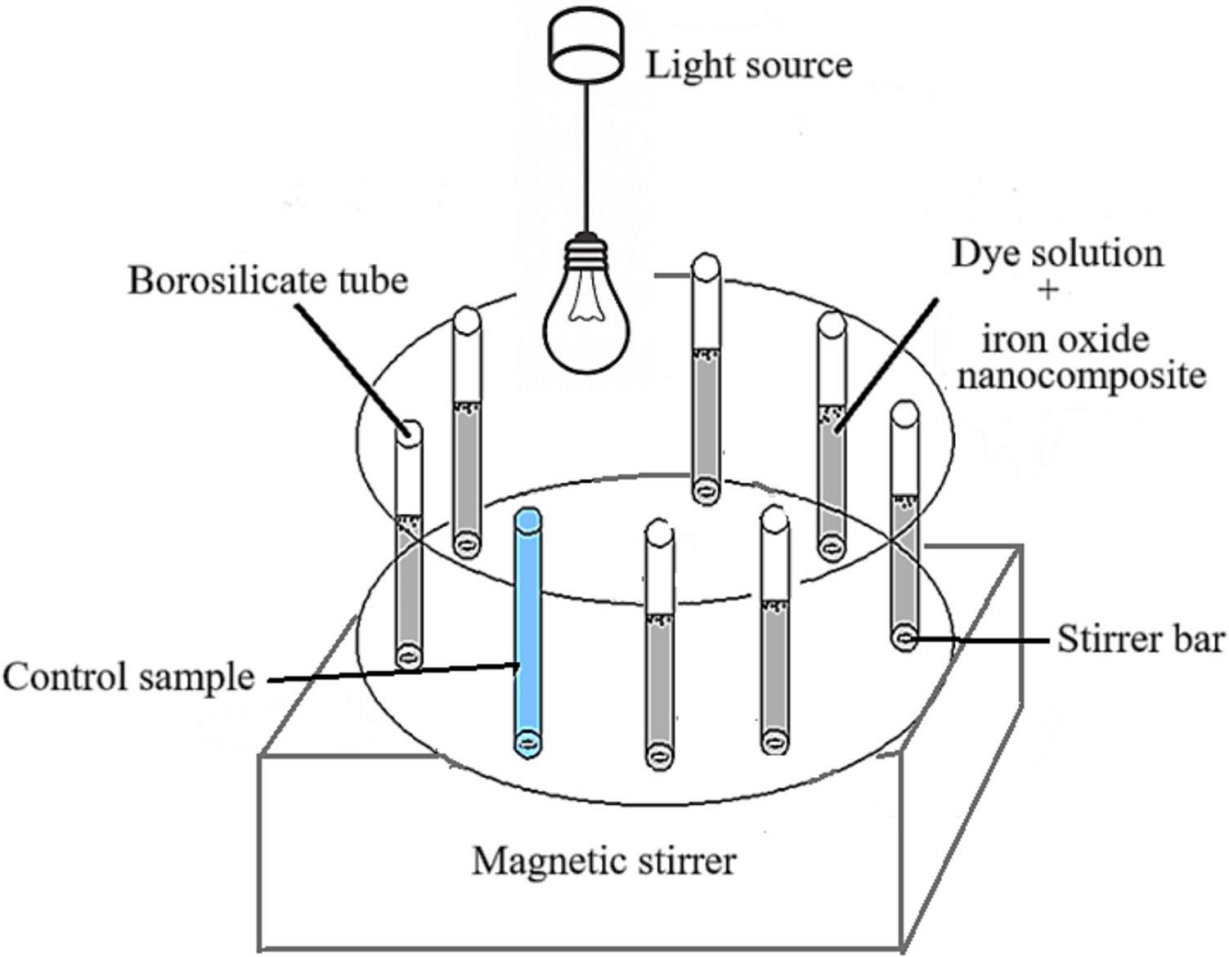
Schematic diagram of the photocatalytic setup

The photocatalytic degradation performance of the iron oxide nanocomposites was assessed by studying the photodegradation of malachite green, rhodamine B, and a binary mixture of malachite green and rhodamine B. Ten parts per million of solutions was prepared for single dyes and 10 ppm of each dye for binary mixtures. Prior to light exposure, the iron oxide nanocomposites and dye solution were stirred in the dark for 30 min to reach adsorption–desorption equilibrium between the dyes and the photocatalyst surface. The experiments were conducted in borosilicate tubes under visible light. For the photodegradation test, 5 mL of a dye solution was mixed with 5 mg of the nanocomposite in seven vials and exposed to an OSRAM HQL (MBF-U) 125 W lamp. A control test without the nanocomposite was also performed to track the dye degradation, as shown in Fig. 1. A vial was removed every 30 min, and the dye concentration was measured using a UV–visible spectrophotometer by recording absorbance in the 200–800 nm range. The degradation efficiency was determined using Eq. (1) (Khakwani et al. 2024):

1 $$D=\frac{{C}_{0} -{ C}_{t}}{{C}_{0} } \times 100$$

where *C*_0_ and *C*_*t*_ represent the dye concentrations prior to and post-light exposure, respectively. The nanocomposite’s photostability and recyclability during photocatalysis were evaluated using the same visible-light exposure. The nanocomposite photocatalysts were separated by an external magnet and rinsed repeatedly with distilled water prior to photodegrading the dyes under the same conditions.

### Detection of reactive species test

One of the key elements of the photocatalytic degradation reaction is the identification of reactive species (Mahboob et al. 2022). Various scavengers, including isopropyl alcohol (IPA), silver nitrate (SN), and benzoquinone (BQ), served as quenching agents for ·OH, e^−^, and ·O_2_^−^, respectively. Each scavenger was introduced into the aqueous malachite or rhodamine B dye solution before the iron oxide nanocomposite was added. The exact procedure was done for aqueous rhodamine B and malachite green binary dye solution.

### Chemical oxygen demand experiments

The COD of the dye solution was determined using the standard dichromate reflux method (Sari et al. 2017). Ten milliliters of the dye-containing sample was transferred into a COD digestion flask. To this, 1.5 mL of 0.25 N potassium dichromate solution was added, followed by 3.5 mL of concentrated sulfuric acid reagent containing silver sulphate as a catalyst. The mixture was gently stirred and heated at 150 °C for 2 h to allow complete oxidation of organic matter. After digestion, the mixture was cooled to room temperature. The remaining dichromate was titrated with a 0.1 N ferrous ammonium sulphate solution using ferroin as an indicator. A blank sample containing all reagents except the organic dye was similarly treated and titrated. The volume of FAS used for both the blank and the sample was recorded and used to calculate the COD value.

## Results and discussion

### Structural studies of iron oxide nanocomposites

**Fig. 2:**
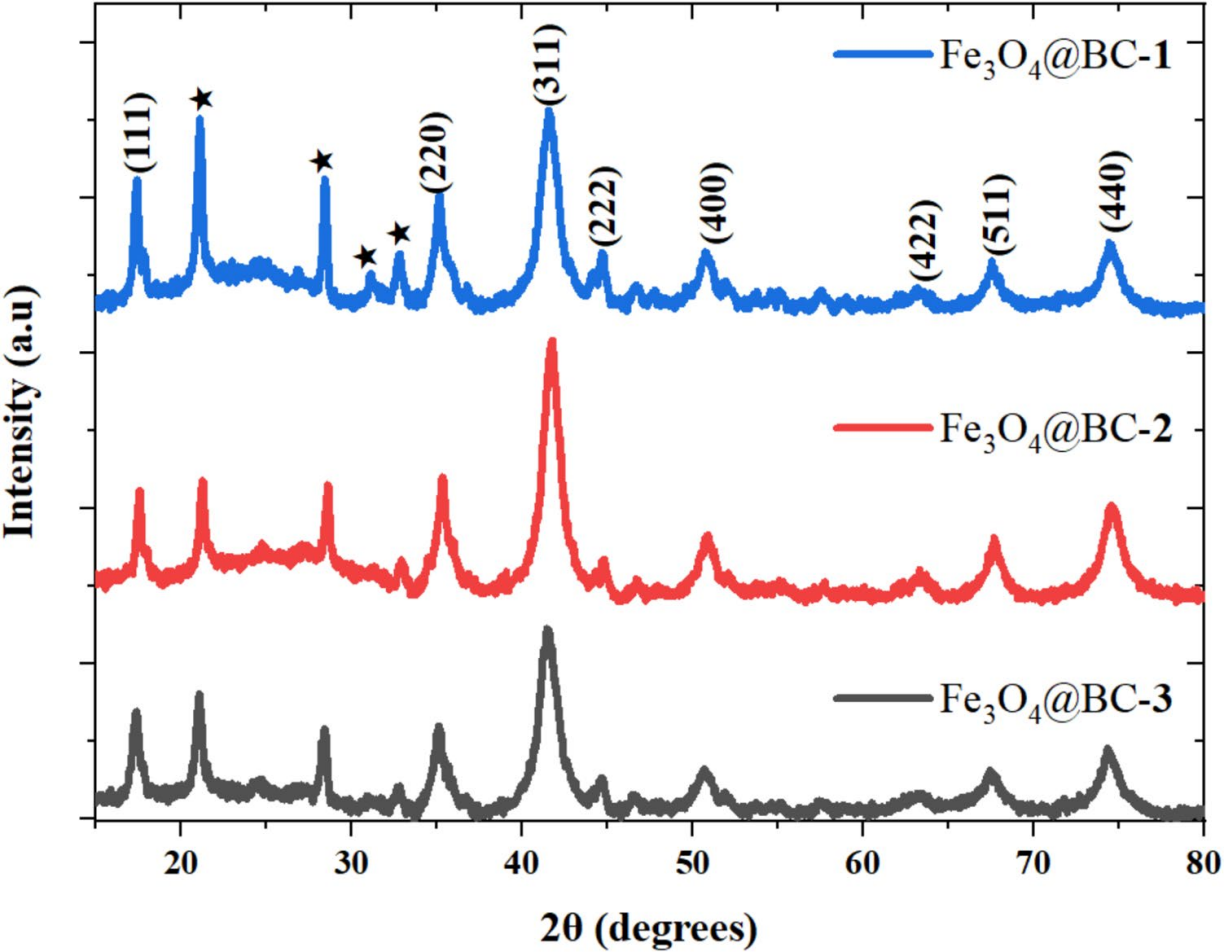
Powder X-ray diffraction patterns of biochar-capped iron oxide nanocomposites

Powder X-ray diffraction (XRD) patterns of the synthesized iron oxide nanocomposites were recorded in the 2θ range of 10° to 80°, as shown in Fig. 2. Reflections along the (111), (220), (311), (222), (400), (422), (511), and (440) planes confirm the crystalline cubic spinel structure of magnetite (PDF ref. 03–065–3107) (Besenhard et al. 2021). The particle sizes of the iron oxide nanocomposites calculated by the Debye–Scherrer equation using the most intense peak were 11.8 nm, 13.5 nm, and 12.2 nm for Fe_3_O_4_@BC–**1**, Fe_3_O_4_@BC–**2**, and Fe_3_O_4_@BC–**3**, respectively. In comparison with the p-XRD pattern of pristine biochar (Fig. S1), the broad amorphous hump and peaks indicated by asterisks observed between 15° and 30° in the iron oxide nanocomposites is attributed to the carbonaceous biochar (Altintig et al. 2018). The intensity of the characteristic (311) peak varied across the patterns, with values of 2645.16 for Fe_3_O_4_@BC–**1**, 3408.04 for Fe_3_O_4_@BC–**2**, and 2457.56 for Fe_3_O_4_@BC–**3**. This variation correlates with the calculated crystallite sizes. The results show that the addition of biochar influences both the peak intensity and crystallite size of the iron oxide nanocomposites (Osadebe et al. 2024).

**Fig. 3:**
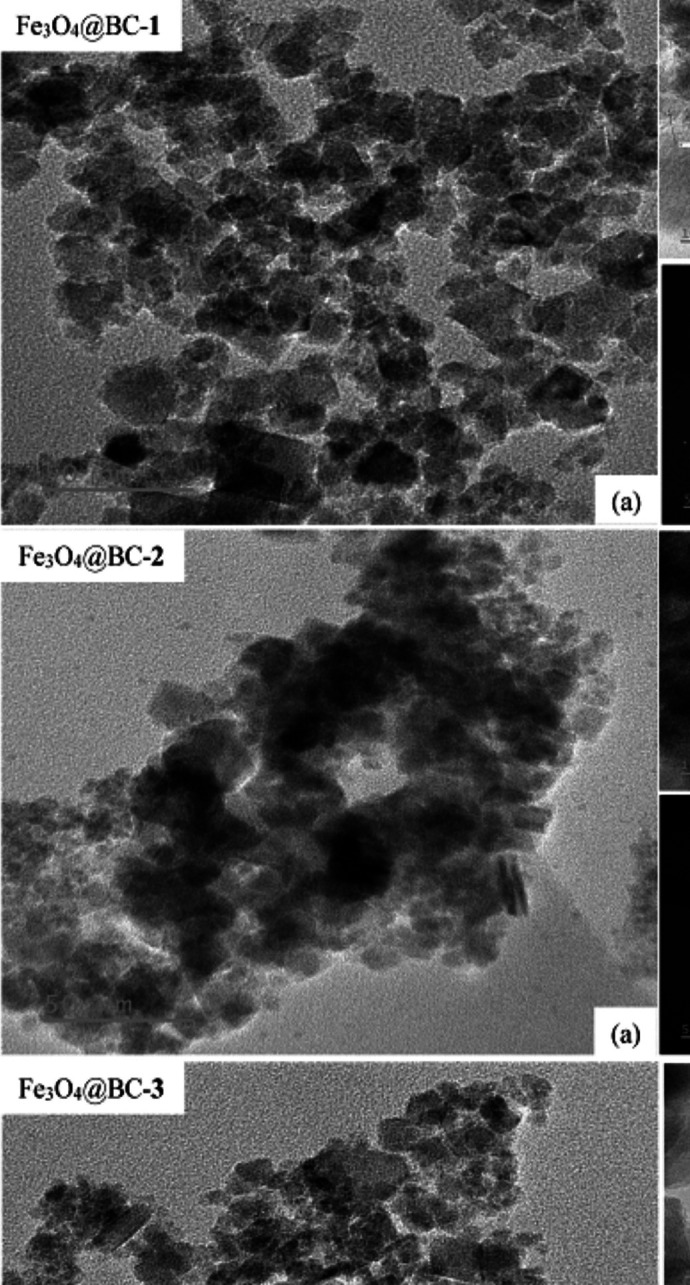
HRTEM micrograph (**a**), lattice fringes (**b**), and SAED (**c**) of biochar-capped iron oxide nanocomposites

HRTEM images of the nanocomposites are presented in Fig. 3a. Fe_3_O_4_@BC–**1** is a mixture of quasi-spherical and square-like particles with a mean particle size of 11.2 nm, while Fe_3_O_4_@BC–**2** comprises agglomerated quasi-spherical, square-like, and rod particles with an average size of 13.3 nm. Fe_3_O_4_@BC–**3** is also a mixture of square, nanorods, and quasi-spherical shaped particles with a mean diameter of 12.9 nm. The particle size distribution histograms of the as-prepared nanocomposites from the HRTEM analysis are presented in Fig. S2. Fe_3_O_4_@BC–**1** exhibits interplanar spacings of 0.285 nm and 0.45 nm, corresponding to the (111) and (311) planes of magnetite, respectively. In contrast, the lattice fringes of Fe_3_O_4_@BC–**2** have interplanar spacings of 0.283 nm and 0.297 nm, while Fe_3_O_4_@BC–**3** shows spacings of 0.289 nm and 0.294 nm, both attributed to the (111) and (220) planes of magnetite (Flores et al. 2024). The concentric rings observed in the SAED patterns (Fig. 3c) are attributed to the polycrystalline nature of the nanocomposites. The well-defined SAED rings and lattice fringes show that the Fe_3_O_4_ nanoparticles retain their crystalline structure within the Fe_3_O_4_@BC nanocomposite. The results suggest that the biochar prepared at different temperatures influences the shapes and particle sizes of the iron oxide nanocomposites.

The XRF analysis of the Fe_3_O_4_@BC–**1**, Fe_3_O_4_@BC–**2**, and Fe_3_O_4_@BC–**3** nanocomposites is presented in Table 1. The results show significant differences in the composites composition influenced by the carbonization temperature of the biochar. The Fe_2_O_3_ content increased with higher carbonization temperatures, ranging from 25.58% in Fe_3_O_4_@BC–**1** (200℃) to 32.39% in Fe_3_O_4_@BC–**3** (600℃), indicating a higher relative proportion of iron oxide at higher carbonization temperatures. The loss on ignition (L.O.I) decreased significantly from 67.98% in Fe_3_O_4_@BC–**1** to 61.13% in Fe_3_O_4_@BC–**3**, indicating the reduction of volatile components as the carbonization temperature increased. This trend suggests that higher carbonization temperatures promote the formation of a more iron-rich composite by minimizing residual organic content and improving phase purity. Trace amounts of other oxides (Al_2_O_3_, K_2_O, MgO, MnO, Na_2_O, P_2_O_5_, SiO_2_, and TiO_2_) were detected with no clear trend observed across the samples which may be due to the retention of mineral content from the *Portulacaria afra* and iron salts precursors.

**Table 1:** X-ray fluorescence (XRF) analysis of Fe_3_O_4_@BC nanocomposites

	Al_2_O_3_	CaO	Cr_2_O_3_	Fe_2_O_3_	K_2_O	MgO	MnO	Na_2_O	P_2_O_5_	SiO_2_	TiO_2_	L.O.I
Fe_3_O_4_–**1**	0.03	2.36	bdl	25.58	0.03	2.65	0.09	0.04	0.31	0.20	0.04	67.98
Fe_3_O_4_–**2**	0.03	3.01	bdl	28.22	0.05	3.15	0.11	0.02	0.46	0.29	0.04	64.82
Fe_3_O_4_–**3**	bdl	2.84	bdl	32.39	0.03	2.81	0.11	0.05	0.49	0.03	0.05	61.13

*bdl* below detection limit

The SEM images of the Fe_3_O_4_@BC nanocomposites revealed notable morphological differences across the three nanocomposites as shown in Fig. S3. Fe_3_O_4_@BC–**1** exhibits a dense granular surface structure, while Fe_3_O_4_@BC–**2** shows a rougher and more porous morphology. Fe_3_O_4_@BC–**3** shows a granule with larger clusters. EDX confirmed the presence of carbon, oxygen, and iron as the predominant elements of the nanocomposites. Minor elements such as magnesium, silicon, and calcium were also detected, which are attributed to the mineral content of the biochar precursor. The highest iron content was recorded for Fe_3_O_4_@BC–**1** (31.54 wt%), corresponding to its dense particle distribution. Fe_3_O_4_@BC–**2** exhibited the highest carbon content (43.80 wt%) and the lowest iron concentration, supporting its more porous surface morphology. Notably, Fe_3_O_4_@BC–**3** contained high oxygen (35.18 wt%). These elemental and morphological variations across the nanocomposites indicate that the synthesis conditions influence the Fe_3_O_4_ nanoparticle dispersion, loading, and surface chemistry, which can influence their photocatalytic activity.

**Fig. 4:**
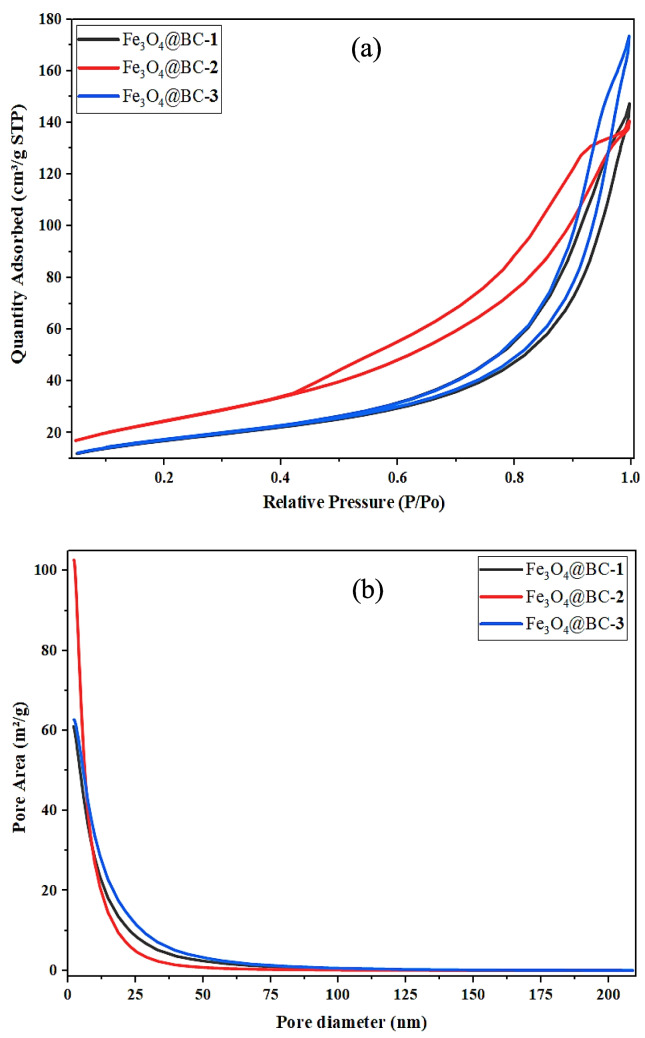
**a** N_2_ adsorption–desorption isotherm and **b** pore size distribution of Fe_3_O_4_@BC nanocomposites

N_2_ adsorption–desorption isotherms of Fe_3_O_4_@BC–**1**, Fe_3_O_4_@BC–**2**, and Fe_3_O_4_@BC–**3** nanocomposites are presented in Fig. 4a. The N_2_ adsorption–desorption isotherms of all three nanocomposites can be classified as type IV, indicating the presence of mesoporous structures (Munonde et al. 2023). The BET surface areas were determined to be 62.3, 91.5, and 63.4 m^2^/g for Fe_3_O_4_@BC–**1**, Fe_3_O_4_@BC–**2**, and Fe_3_O_4_@BC–**3**, respectively. Fe_3_O_4_@BC–**2** exhibited the highest surface area, which can be attributed to its more developed microporous and small mesoporous structure, enhancing the availability of active sites for adsorption and catalytic processes (Ozcan et al. 2024). The average pore diameters for Fe_3_O_4_@BC–**1**, Fe_3_O_4_@BC–**2**, and Fe_3_O_4_@BC–**3** are 14.2 nm, 8.9 nm, and 16.7 nm, respectively, which indicate that all nanocomposites are predominantly mesoporous with a certain contribution from their microporosity (Sobrinho et al. 2019). This observation is further confirmed by the pore size distributions depicted in Fig. 4b, which reveal that Fe_3_O_4_@BC–**3** possesses larger mesopores, while Fe_3_O_4_@BC–**2** contains relatively smaller mesopores. In addition, Fe_3_O_4_@BC–**3** showed the highest cumulative pore volume (0.2624 cm^3^/g), indicating a more porous network compared to Fe_3_O_4_@BC–**1** (0.2174 cm^3^/g) and Fe_3_O_4_@BC–**2** (0.2282 cm^3^/g). The porous nature of Fe_3_O_4_@BC–**3** facilitates enhanced mass transport of larger molecules, whereas the higher surface area and smaller mesopores of Fe_3_O_4_@BC–**2** suggest improved accessibility and surface interaction (Wang et al. 2019).

The FTIR spectra of Fe_3_O_4_@BC nanocomposites (Fig. S4(a)) showed characteristic peaks that correspond to the O–H stretching and H–O–H bending vibrations at 3400 cm^−1^ and 1630 cm^−1^, respectively, which confirms the presence of surface hydroxyl groups and adsorbed water. Peaks in the region 1400–1600 cm^−1^ were attributed to C = C or aromatic ring vibrations from the biochar matrix, while bands around 1100 cm^−1^ suggested C–O or C–OH stretching vibrations. A strong peak at 550 cm^−1^ confirmed the presence of Fe–O bonds from the Fe_3_O_4_ (Ananthi et al. 2022). After photocatalysis (Fig. S4(b)), changes observed included reduced intensity and change in shape of the O–H band, and the emergence of peaks in the 1400–1600 cm^−1^ range, which indicates adsorption of dye residues or degradation intermediates.

The Fe–O peak remained prominent which confirms the structural integrity of the Fe_3_O_4_ core post-photocatalysis. The spectral changes observed suggest that the photocatalytic process induced surface modifications while preserving the iron oxide nanoparticles.

### Optical studies of iron oxide nanocomposites

The absorption spectra of the iron oxide nanocomposites are shown in Fig. S5(a). The absorption peak for Fe_3_O_4_@BC–**1** is observed at 331 nm, for Fe_3_O_4_@BC–**2** at 351 nm, and for Fe_3_O_4_@BC–**3** at 296 nm. The sharp absorption edges indicate a well-defined band structure with reduced mid-gap defect states (Bahadur et al. 2017). Tauc plots, shown in Fig. S5(b), were used to estimate optical bandgap energies by plotting (αhυ)^2^ against (hυ), assuming a direct allowed transition. The calculated optical bandgaps are 1.85 eV, 1.79 eV, and 1.97 eV for Fe_3_O_4_@BC–**1**, Fe_3_O_4_@BC–**2**, and Fe_3_O_4_@BC–**3**, respectively. The notable differences in the optical bandgaps of the nanocomposites prepared with biochar carbonized at different temperatures suggest that biochar plays a key role in tuning the optical properties of the resulting iron oxide nanocomposites.

### Photocatalytic degradation of malachite green and rhodamine B dyes by the biochar-capped iron oxides nanoparticles

#### Effect of irradiation time on photocatalytic degradation

**Fig. 5:**
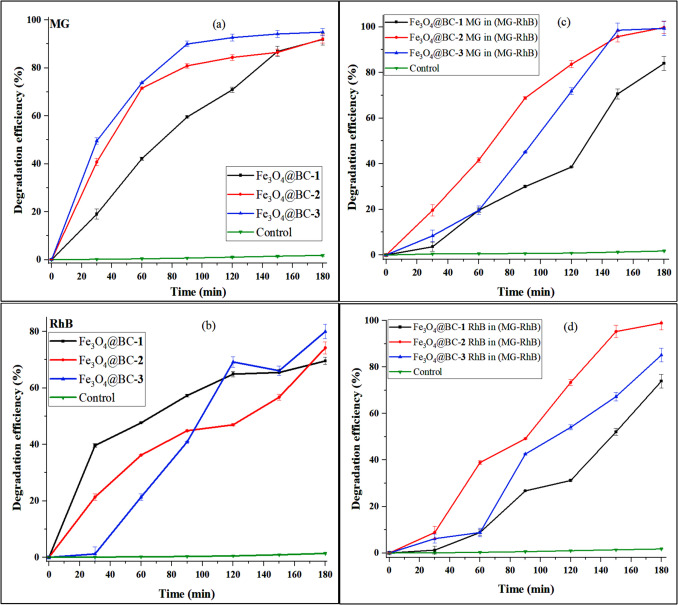
Degradation curve of malachite green and rhodamine B single dyes (**a**, **b**) and malachite green and rhodamine B binary dyes using Fe_3_O_4_@BC nanocomposites (**c**, **d**)

The photocatalytic potential of the iron oxide nanocomposites was evaluated against single dye solutions of MG, RhB, and a binary mixture of the two dyes (MG–RhB) under visible light. The absorption spectra of the MG and RhB binary solution showed no overlap between the two dyes, and the absorption maxima of the dyes decreased with increased visible light exposure (Fig. S6-8). The disappearance of the malachite green chromophore peak around 617 nm in the UV–Vis spectrum suggests that the dye has undergone significant decolorization, and the chromophore structure has been broken down. However, the appearance of peaks below 400 nm could suggest the formation of intermediate compounds during the degradation process. The degradation efficiencies for Fe_3_O_4_@BC–**1**, Fe_3_O_4_@BC–**2**, and Fe_3_O_4_@BC–**3** against MG were 91.91%, 92.06%, and 94.91%, respectively (Fig. 5a). For RhB, the degradation efficiencies were 69.57%, 74.20%, and 80.01% for Fe_3_O_4_@BC–**1**, Fe_3_O_4_@BC–**2**, and Fe_3_O_4_@BC–**3** after 180 min (Fig. 5b). These results indicate that Fe_3_O_4_@BC–**3** is the most efficient photocatalyst for the degradation of both MG and RhB single dyes. The superior photocatalytic performance of Fe_3_O_4_@BC–**3** can be attributed to its larger cumulative pore volume and larger mesopores, which facilitate enhanced mass transport of dye molecules within its porous network (Wang et al. 2019). The synergistic interaction between biochar and iron oxide nanoparticles further enhances electron transfer and photocatalytic efficiency (Cheng et al. 2022).

In the MG–RhB mixture, the photodegradation efficiency of MG was 83.95% with Fe_3_O_4_@BC–**1**, 99.74% with Fe_3_O_4_@BC–**2**, and 99.33% with Fe_3_O_4_@BC–**3**. For RhB, the degradation efficiency was 73.92% with Fe_3_O_4_@BC–**1**, 98.89% with Fe_3_O_4_@BC–**2**, and 85.17% with Fe_3_O_4_@BC–**3**. Overall, Fe_3_O_4_@BC–**2** demonstrated superior photocatalytic performance for the mixed dye system compared to the other nanocomposites. The enhanced activity of Fe_3_O_4_@BC–**2** can be attributed to its high surface area, providing a higher density of accessible active sites that can improve adsorption and catalytic interactions. Additionally, biochar serves as an effective electron reservoir and acceptor, preventing electron–hole recombination and extending the lifespan of reactive species (Rangarajan et al. 2022; Amdeha 2023; Huang et al. 2024). As a result, the biochar coating on the nanoparticles ensures sustained photocatalytic activity, making it a crucial component in photocatalytic systems.

To determine the most appropriate kinetic model for the photocatalytic degradation of the dyes, the experimental data were fitted to pseudo-first-order, pseudo-second order, and Langmuir–Hinshelwood models. The corresponding *R*^2^ values and residual sum of squares (RSS) are summarized in Table S1, while the residual plots are presented in Figs. S10–S13. Among the models tested, the pseudo-first-order model exhibited the highest *R*^2^ values and the lowest RSS for both dyes across all photocatalysts, which indicates a better fit. The residual plots for the pseudo-first-order model also showed randomly distributed and minimal errors compared to those from the pseudo-second order and Langmuir–Hinshelwood models. Fig. S9 presents the natural logarithm of *C*_*t*_/*C*_0_ versus irradiation time which shows a linear relationship (Halomoan et al. 2022). RhB and MG degrade at different rates due to the difference in their affinity for the catalyst surface and their chemical structures. The highest rate constants of MG and RhB single dyes are 0.01728 and 0.00942 min^−1^ obtained from Fe_3_O_4_@BC–**3**, whereas the rate constants of 0.01648 and 0.01346 min^−1^ were obtained using Fe_3_O_4_@BC–**3** for MG and RhB in the binary mixture. The rate constants and *R*^2^ values of the pseudo-first-order kinetic model are summarized in Table 2.

**Table 2:** The photocatalytic degradation parameters of single and mixed dyes over biochar-capped iron oxides nanoparticles under visible light irradiation

Dyes	Photocatalysts	Degradation efficiency (%)	Rate constant *k* (min^−1^)	*R*^2^
MG	Fe_3_O_4_@BC-**1**	91.91	0.01413	0.9819
	Fe_3_O_4_@BC-**2**	92.06	0.01328	0.9758
	Fe_3_O_4_@BC-**3**	94.91	0.01728	0.9655
RhB	Fe_3_O_4_@BC-**1**	69.57	0.00606	0.9505
	Fe_3_O_4_@BC-**2**	74.20	0.00648	0.9644
	Fe_3_O_4_@BC-**3**	80.01	0.00942	0.9650
MG in (MG–RhB)	Fe_3_O_4_@BC-**1**	83.95	0.00968	0.9191
	Fe_3_O_4_@BC-**2**	99.74	0.01648	0.9917
	Fe_3_O_4_@BC-**3**	99.33	0.01208	0.9579
RhB in (MG–RhB)	Fe_3_O_4_@BC-**1**	73.92	0.00686	0.9147
	Fe_3_O_4_@BC-**2**	98.89	0.01346	0.9579
	Fe_3_O_4_@BC-**3**	85.14	0.01013	0.9447

The degradation efficiency of malachite green (MG) and rhodamine B (RhB) dyes by the Fe_3_O_4_@BC nanocomposites was compared to that of other catalysts used in earlier studies as displayed in Table 3. The results demonstrate the photocatalytic superiority of the Fe_3_O_4_@BC nanocomposites over other photocatalysts used in previous studies because they degrade the dyes using a small amount of catalyst in a short period of time with high efficiency. Several other studies have demonstrated the effectiveness of Fe_3_O_4_-biochar composites in the photocatalytic degradation of organic pollutants, showing biochar’s role as a support material. For instance, an ultrasonic-assisted Fe_3_O_4_/rice husk biochar photocatalyst demonstrated a degradation efficiency of 96.7% for ciprofloxacin under UVA irradiation after 180 min, attributed to enhanced surface area and improved charge separation (Toan et al. 2023). Similarly, a hydrothermally synthesized Fe_3_O_4_-biochar composite derived from avocado peel exhibited rapid adsorption kinetics and high removal efficiency (~ 89%) for methylene blue, highlighting the advantage of biochar as a support material (Prabakaran et al. 2022). In another study, a magnetic nano-β-FeOOH/Fe_3_O_4_/biochar composite showed significant enhancement in the photocatalytic degradation of methyl orange dye, which was due to synergistic effects between iron oxides and biochar that facilitate electron–hole separation and reduce recombination (Zhang et al. 2021). Surface modification of biochar with polyoxometalates in a PW_12_/Fe_3_O_4_/biochar nanocomposite significantly enhanced metronidazole removal, demonstrating the critical role of surface chemistry in optimizing catalytic efficiency (Mohammadian et al. 2024). In contrast to previous studies, our Fe_3_O_4_@BC–**2** composite, synthesized using *Portulacaria afra* derived biochar at 400 °C, achieved high photocatalytic degradation efficiencies, reaching 99.74% for malachite green and 98.89% for rhodamine B within a binary dye system. This photocatalytic activity outperforms comparable systems demonstrating superior applicability for complex wastewater treatment. Furthermore, while prior studies focused on single dye systems or required additional surface modifications, the current study introduces a novel, cost-effective, and sustainable biochar precursor with tunable properties through carbonization temperature to enhance photocatalytic activity of the iron oxide nanocomposite. These results demonstrate the distinctive advantages of *Portulacaria afra* derived biochar, showing the role of precursor selection and carbonization temperature influence in Fe_3_O_4_@BC nanocomposite optimization for practical wastewater treatment applications.

**Table 3:** Comparison of photocatalytic activity of the Fe_3_O_4_@BC nanocomposites with other catalysts in literature

Dyes	Photocatalyst	Catalyst dosage (mg)	Dye concentration (ppm)	Time (min)	Degradation efficiency (%)	Reference
MG	CuO-Gd_2_Ti_2_O_7_	10	5	90	88.60	Halomoan et al. (2022)
	BiOBr/Ag_3_PO_4_	-	10	200	93.44	Kokilavani et al. (2022)
	CH/Ce–ZnO	5	5	270	87	Saad et al. (2020)
	SnO_2_/ZnO	10	10	150	98	Zhang et al. (2022)
	Fe_3_O_4_@BC–**3**	5	10	180	94.91	**This study**
RhB	CuS/ZnS	10	5	270	97	Mugumo et al. (2023)
	PbCrO_4_/ZnO	100	5	180	77	Hamza et al. (2022)
	BiMnO_3_	20	5	150	68	Revathi et al. (2020)
	TiO_2_	5	10	180	20.5	Suhaimi et al. (2022)
	Fe_3_O_4_@BC–**3**	5	10	180	80.01	**This study**
MG in (MG–RhB) RhB in (MG–RhB)	Fe_3_O_4_@BC–**2**	5	10	180	99.74	**This study**
	Fe_3_O_4_@BC–**2**	5	10	180	98.89	**This study**

#### Effect of scavengers on the photocatalytic degradation efficiency of the dyes

The ability of a photocatalyst to produce long-lived electron–hole pairs, which enable redox reactions to release active species like superoxide and hydroxyl radicals, regulates the efficiency of photocatalytic reactions. To identify the reactive species responsible for photodegradation, isopropyl alcohol (IPA), benzoquinone (BQ), and silver nitrate (SN) were used as scavengers to neutralize ·OH, ·O_2_^−^, and e^−^, respectively (Schneider et al. 2020; Ge et al. 2022; Rout et al. 2022). Fig. S14 illustrates the scavenging activity of the nanocomposites on both single and mixed dyes. A comparison of the photodegradation of malachite green, rhodamine B, and the binary dye solution, after adding scavengers, shows that the degradation efficiencies of malachite green by Fe_3_O_4_@BC–**1** drops from 91.91 to 22.16% (IPA), 40.90% (SN), and 34.91% (BQ). Similarly, the degradation efficiencies by Fe_3_O_4_@BC–**2** decreases from 92.06 to 29.01%, 41.56%, and 36.56%, and for Fe_3_O_4_@BC–**3**, it decreased from 94.91 to 17.77%, 33.84%, and 27.54%. This suggests that ·OH and ·O_2_^−^ are the dominant active species, while e^−^ plays a secondary role, which is consistent with results from other studies (Mohanty et al. 2022; Mostafa and Amdeha 2022; Sobhani 2024). For rhodamine B, the degradation efficiency of Fe_3_O_4_@BC–**1** decreases from 69.51 to 16.96% (IPA), 33.75% (SN), and 24.2% (BQ). For Fe_3_O_4_@BC–**2**, it decreases from 74.20 to 6.20% (IPA), 37.87% (SN), and 20.87% (BQ), while for Fe_3_O_4_@BC–**3**, it decreases from 80.01 to 19.23%, 36.14%, and 23.01%. For MG dye in the mixed dyes, the degradation efficiency decreases from 83.95 to 29.98%, 42.61%, and 33.57% by Fe_3_O_4_@BC–**1**. With Fe_3_O_4_@BC–**2**, it decreases from 99.74 to 19.61%, 32.83%, and 21.65%, while for Fe_3_O_4_@BC–**3**, the efficiency decreases from 99.33 to 8.41%, 35.11%, and 19.61%. Similar trends were observed for rhodamine B degradation in the binary dye mixture after the addition of scavenger. With the binary dyes, the addition of IPA, SN, and BQ reduces RhB degradation in the MG-RhB mixture from 73.92 to 8.83%, 31.19%, and 26.72% by Fe_3_O_4_@BC–**1**. For Fe_3_O_4_@BC–**2**, the degradation efficiency decreases from 98.89 to 8.36%, 39.21%, and 28.93%, and for Fe_3_O_4_@BC–**3**, it drops from 85.14 to 6.18%, 32.56%, and 8.83%. These results highlight that ·OH and ·O_2_^−^ play crucial roles in the degradation of RhB, which is also consistent with findings from other photocatalysts (Mohanty et al. 2022; Mzimela et al. 2022; Mane et al. 2024). The presence of BQ and IPA significantly reduces degradation efficiency, demonstrating the importance of ·O₂^−^ and ·OH radicals in the reaction. Scavenging these radicals inhibits the degradation process. BQ’s effect on degrading efficiency implies that superoxide radicals are crucial, although less critical than hydroxyl radicals. Among scavengers, IPA has a major impact since it targets hydroxyl radicals, which are the main species that break down MG and RhB when using the Fe_3_O_4_@BC nanocomposites.

**Scheme 1:**
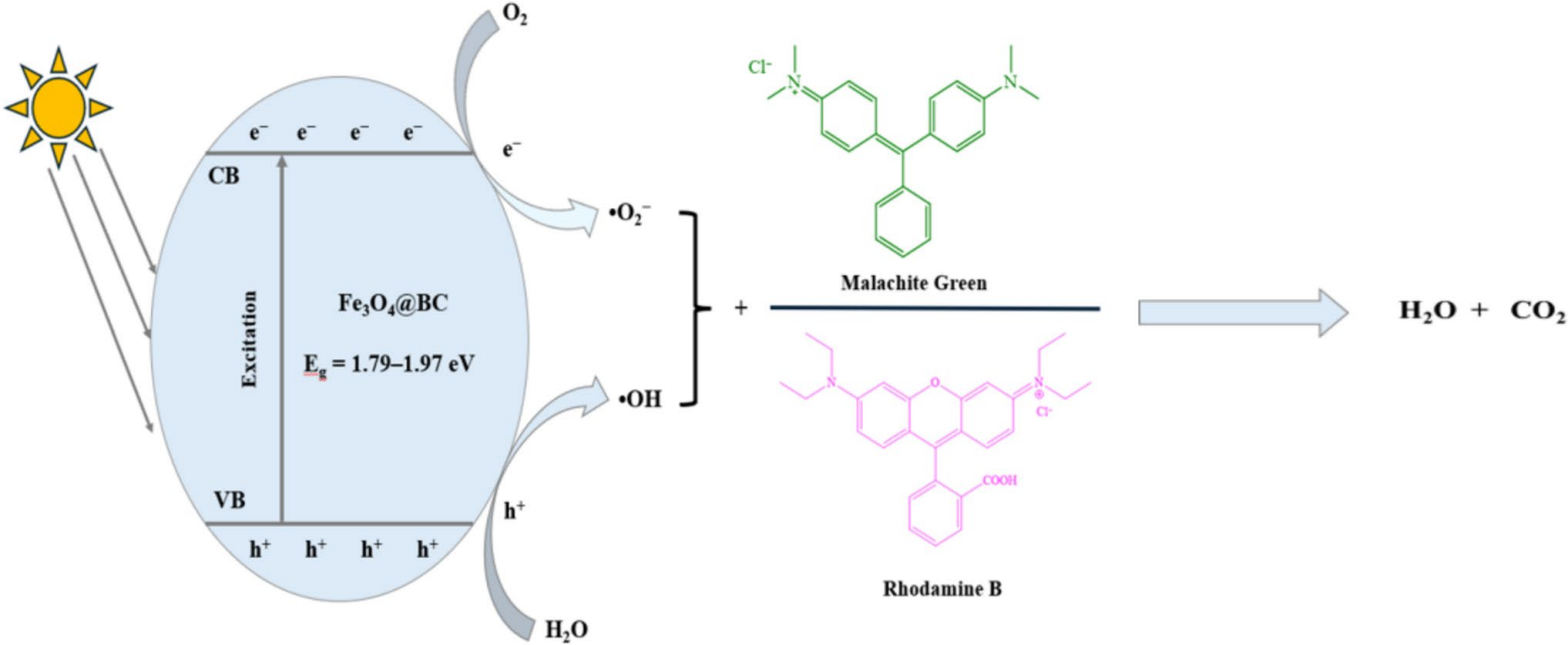
Schematic illustration of the photocatalytic degradation mechanism

Scheme 1 illustrates the proposed mechanism for the degradation of malachite green and rhodamine B using Fe_3​_O_4_​@BC nanocomposites. When the catalyst absorbs photons, electrons are excited from the valence band to the conduction band, leading to the formation of electron–hole (e^−^/h^+^) pairs in the conduction and valence bands, respectively. These photogenerated electrons and holes participate in oxidation and reduction reactions during the photocatalytic process, resulting in the production of hydroxyl radicals (·OH) and superoxide radicals (·O_2_^−^). These reactive species play a key role in breaking down complex organic pollutants into smaller, non-toxic molecules, eventually leading to their mineralization. The charge transfers involved are shown in Eqs. 2–5 (Jakimińska et al. 2022; Jabeen et al. 2023):

2 $$\text{Fe}_3\text{O}_4@\text{BC}+\text{h}\nu\rightarrow\text{h}^++\text{e}^-$$

3 $$\mathrm e^-\:+\:{\mathrm O}_2\:\rightarrow\:\cdot\mathrm O_2^-$$

4 $$\mathrm h^+\;\:+\:{\mathrm H}_2\mathrm O\:\rightarrow\:\cdot\mathrm{OH}\:+\:\mathrm H^+$$

5 $$\cdot{\mathrm O}_2^-\;\:+\:\cdot\mathrm{OH}\:+\:\mathrm{dyes}\:\rightarrow\:\:\rightarrow\:\mathrm{Intermediates}\:\rightarrow\:{\mathrm{CO}}_2\:+\:{\mathrm H}_2\mathrm O\;$$

#### Effect of pH on photocatalytic degradation of the dyes

**Fig. 6:**
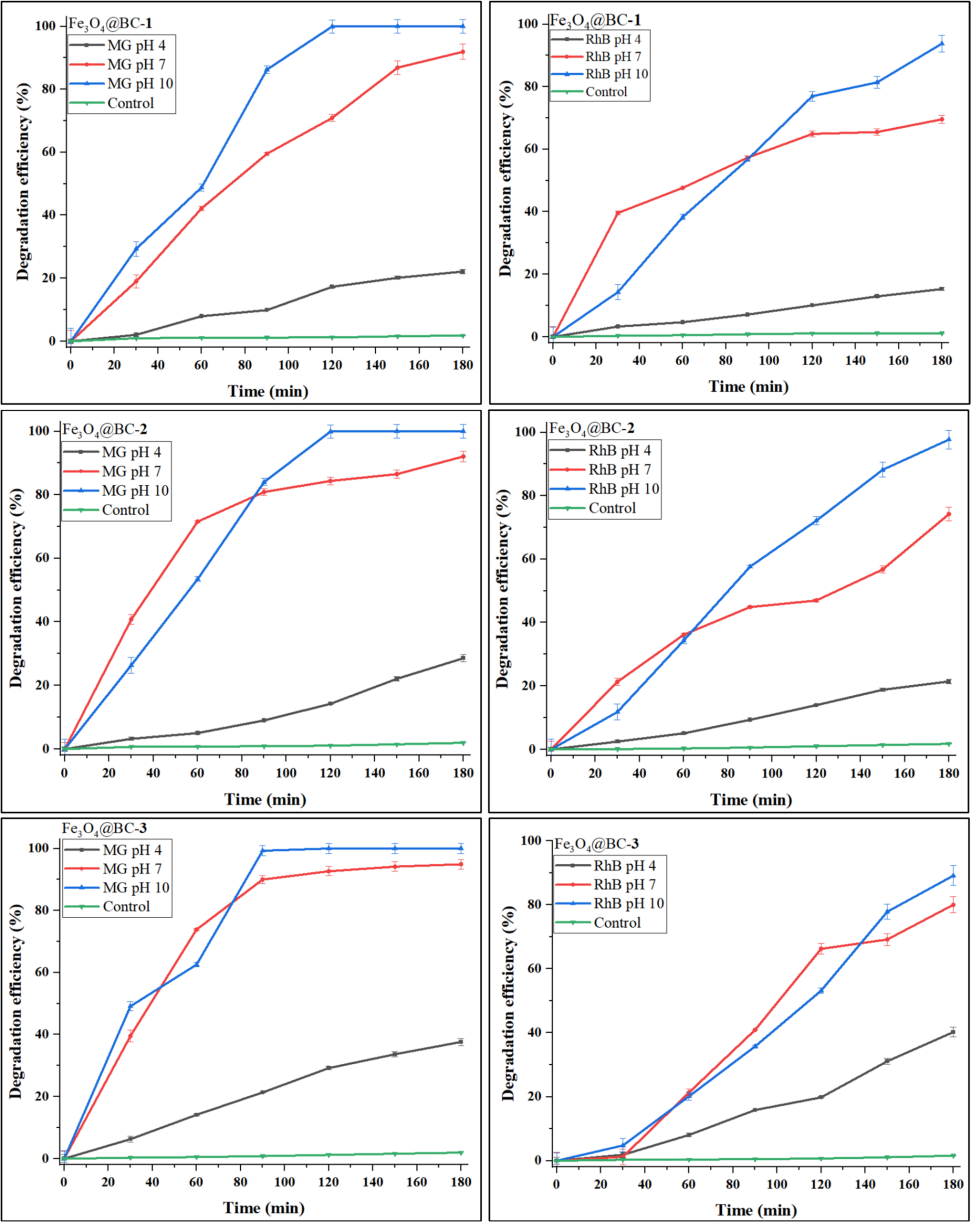
Effect of pH on malachite green and rhodamine B single dyes over Fe_3_O_4_@BC nanocomposites

**Fig. 7:**
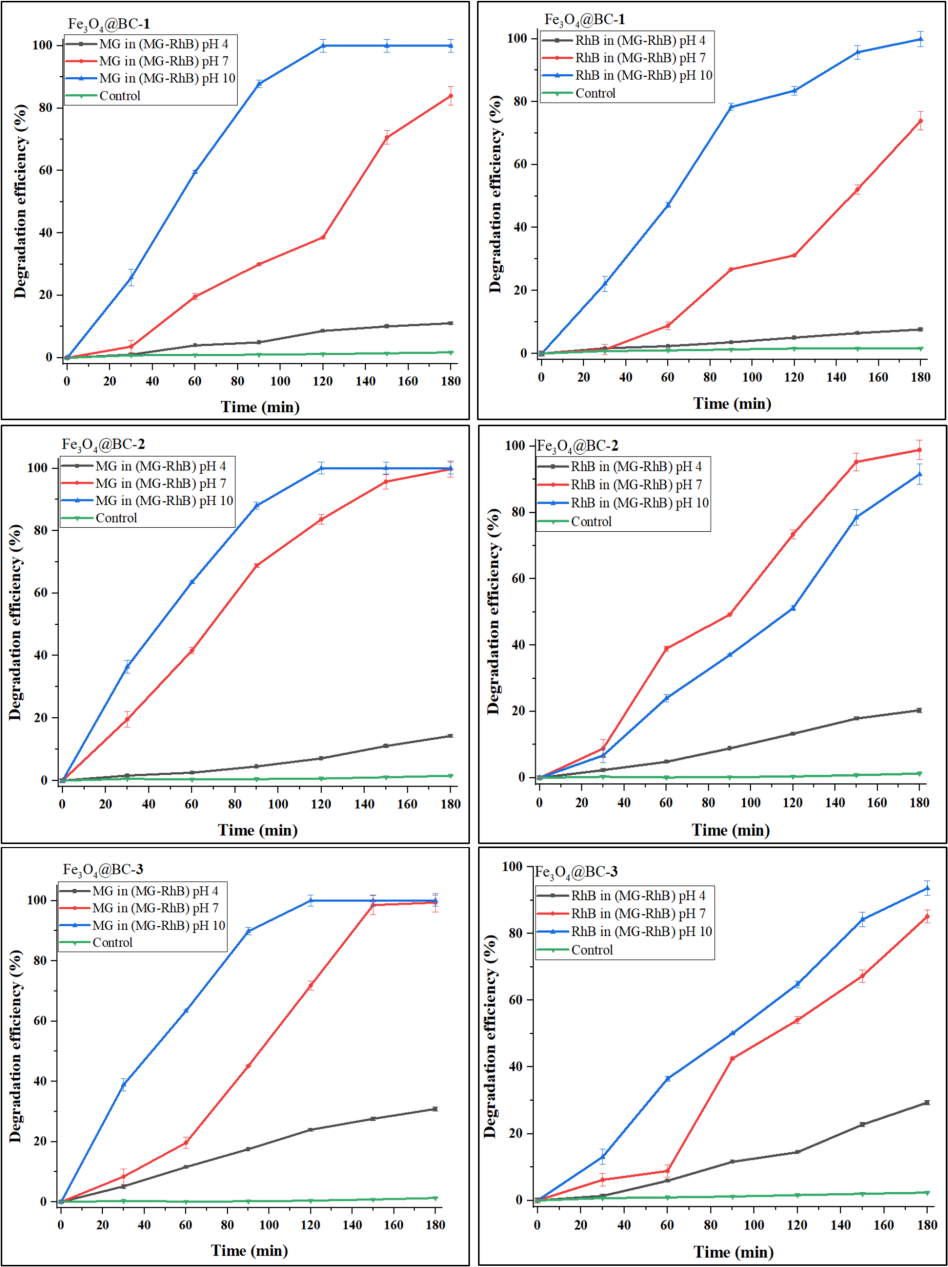
Effect of pH on malachite green and rhodamine B in malachite green-rhodamine B binary dyes over Fe_3_O_4_@BC nanocomposites

It is essential to assess photocatalytic efficiency at different pH levels, as pH significantly influences both the catalyst’s surface charge and the properties of the organic dye (Farahbakhsh et al. 2022; Bazrafshan et al. 2023). Figure 6 illustrates the effect of pH on single organic dyes and Fig. 7 shows the effect of pH on binary dye mixtures. Influence of pH on the photocatalytic degradation of the dyes were studied at pH levels (4, 7, and 10) using a constant catalyst dosage and dye concentration for MG, RhB, and MG–RhB solutions. The results show that degradation efficiencies for MG and RhB decreased in acidic medium but increased in basic medium, which indicates that H^+^ and OH^−^ ions play a crucial role in the photocatalytic process. In acidic media, H⁺ concentration increases, while OH⁻ concentration rises in basic media. At pH 4, RhB degradation efficiency dropped to 15.28% for Fe_3_O_4_@BC–**1**, 21.45% for Fe_3_O_4_@BC–**2**, and 40.21% for Fe_3_O_4_@BC–**3**. In contrast, at pH 10, the degradation efficiency improved to 93.78% for Fe_3_O_4_@BC–**1**, 97.70% for Fe_3_O_4_@BC–**2**, and 89.11% for Fe_3_O_4_@BC–**3**. Rhodamine B tends to self-aggregate in acidic media, forming dimers or larger aggregates, which hinder its interaction with reactive species, reducing photocatalytic efficiency (Fanciullo et al. 2023).

At higher pH, RhB exists primarily in its monomeric form, which allows better interactions with the photocatalyst and reactive species, and consequently higher degradation efficiencies. The degradation efficiency of malachite green at pH 4 was 22.12% for Fe_3_O_4_@BC–**1**, 28.58% for Fe_3_O_4_@BC–**2**, and 37.63% for Fe_3_O_4_@BC–**3**, while complete degradation was observed at pH 10. A similar trend was noted for the binary dye mixture (Fig. 7). The photocatalytic degradation efficiency for MG at lower pH is significantly reduced due to competition with H_3_O^+^ ions, which can block a substantial number of active sites (Hassan et al. 2023). As the pH increased from 4 to 10, degradation efficiency increased for all nanocomposites due to the absence of H_3_O^+^ competing ions, allowing the positively charged MG to be easily attracted to the free active sites on the catalyst surface.

#### Chemical oxygen demand studies

The photocatalytic activity of the Fe_3_O_4_@BC nanocomposites was further evaluated by measuring chemical oxygen demand (COD) removal. Fig. S15 shows the COD removal efficiencies for malachite green (MG) ranging from 62.57% for Fe_3_O_4_@BC–**1** to 75.04% for Fe_3_O_4_@BC–**3**, while for rhodamine B (RhB) they ranged from 58.08 to 72.02%. In the binary dye system, COD removal reached the highest efficiency of 84.35% with Fe_3_O_4_@BC–**2**. These results indicate significant mineralization of organic dyes, with removal trends that correlate with those observed in the dye degradation efficiency curves.

#### Photostability studies of the biochar-capped iron oxide nanoparticles

**Fig. 8:**
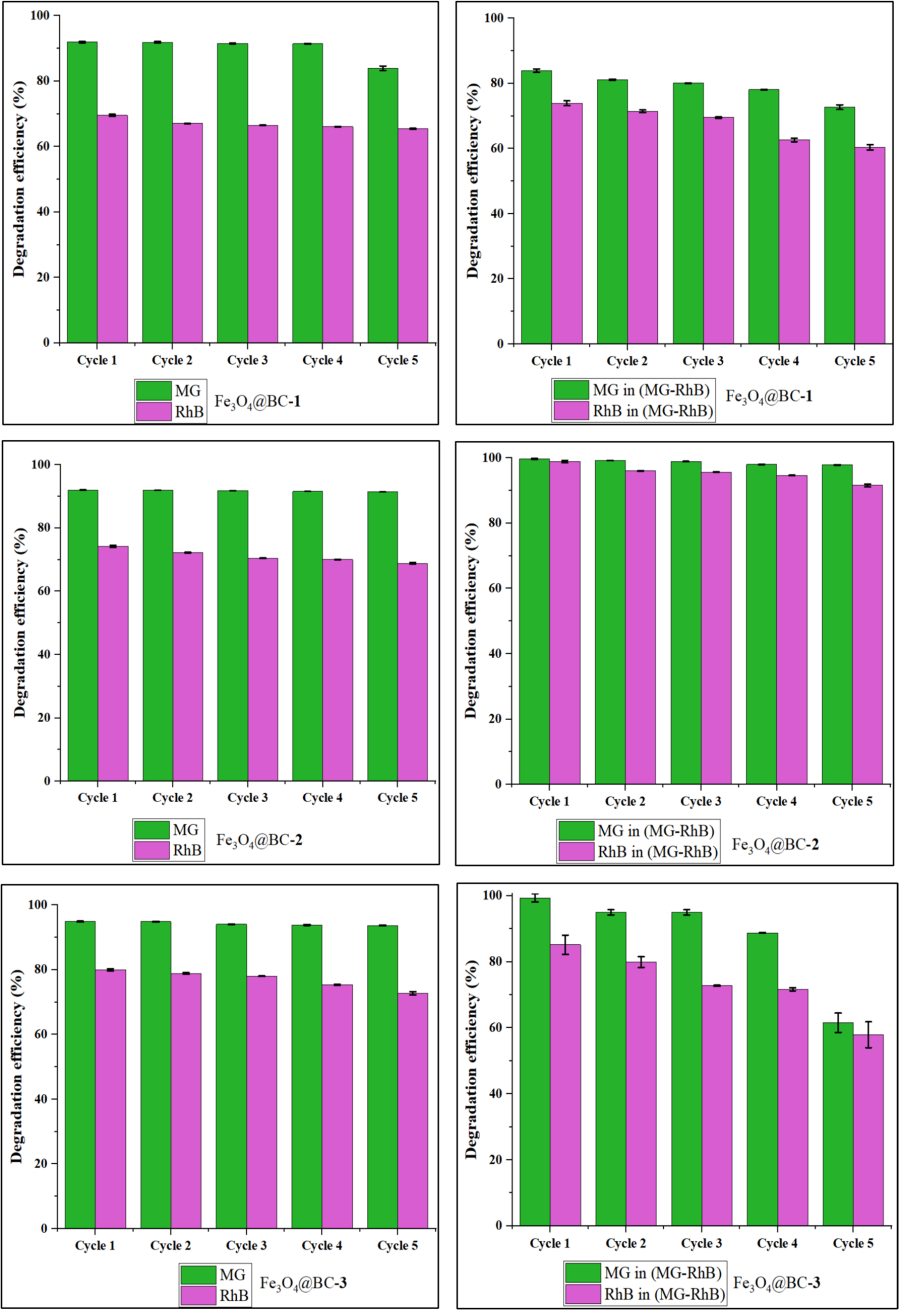
Reusability test of as-prepared Fe_3_O_4_@BC catalysts on MG, RhB, and binary dyes

The reusability and photostability of the synthesized iron oxide nanocomposites as photocatalysts are crucial for practical applications. To assess their stability, the nanocomposites were subjected to five consecutive photocatalytic cycles under the same reaction conditions. As illustrated in Fig. 8, the degradation efficiencies of the nanocomposites exhibited minimal changes up to the fourth cycle. After five cycles, the degradation efficiencies for malachite green were 83.95%, 91.46%, and 93.69% for Fe_3_O_4_@BC–**1**, Fe_3_O_4_@BC–**2**, and Fe_3_O_4_@BC–**3**, respectively, while for rhodamine B, the degradation efficiencies were 65.40%, 68.87%, and 72.73%. The nanocomposites also demonstrated strong reusability and stability for the degradation of binary dyes. After five cycles, the degradation efficiencies for malachite green in the binary dye system were 72.73%, 97.90%, and 61.52% for Fe_3_O_4_@BC–**1**, Fe_3_O_4_@BC–**2**, and Fe_3_O_4_@BC–**3**, while for rhodamine B, they were 60.39%, 91.56%, and 57.89%, respectively.

## Conclusions

*Portulacaria afra* was carbonized at 200℃ to prepare Fe_3_O_4_@BC–**1**, at 400℃ to prepare Fe_3_O_4_@BC–**2**, and 600℃ to prepare Fe_3_O_4_@BC–**3** iron oxide nanocomposites. Powder X-ray diffraction analysis revealed the cubic magnetite (Fe_3_O_4_) crystalline phase of iron oxide. HRTEM images showed rods, spherically shaped and square-like particles with particle sizes of 11.2 nm for Fe_3_O_4_@BC–**1**, 13.3 nm for Fe_3_O_4_@BC–**2**, and 12.9 nm for Fe_3_O_4_@BC–**3**. BET analysis confirmed mesoporous structures in all Fe_3_O_4_@BC samples. Fe_3_O_4_@BC–**2** exhibited the highest surface area of 91.5 m^2^/g, while Fe_3_O_4_@BC–**3** showed the largest pore volume (0.2624 cm^3^/g) and diameter (16.7 nm). The energy band gap of the iron oxide nanocomposites was estimated between 1.79 eV and 1.97 eV. The iron oxide nanocomposites were used as photocatalysts for the degradation of malachite green (MG), rhodamine B (RhB), and a mixture of malachite green and rhodamine B binary dyes (MG–RhB). Photocatalytic degradation efficiencies of 91.91% (Fe_3_O_4_@BC–**1**), 92.06% (Fe_3_O_4_@BC–**2**), and 94.91% (Fe_3_O_4_@BC–**3**) were obtained for MG, while degradation efficiencies of 69.57% (Fe_3_O_4_@BC–**1**), 74.20% (Fe_3_O_4_@BC–**2**), and 80.01% (Fe_3_O_4_@BC–**3**) were obtained for RhB. In the binary dye system, malachite green degradation reached 83.95% with Fe_3_O_4_@BC–-**1**, 99.74% with Fe_3_O_4_@BC–**2**, and 99.33% with Fe_3_O_4_@BC–-**3**, while rhodamine B degradation was 73.92%, 98.89%, and 85.14%, respectively. This study shows the potential of *Portulacaria afra* as a sustainable biochar precursor in which the biochar synergistically interacts with iron oxide nanoparticles and enhances its photocatalytic activity. Hydroxyl radicals and superoxide anions were identified as the primary active species in the photodegradation process. The iron oxide nanocomposites exhibited enhanced photocatalytic efficiency in basic media. Reusability tests demonstrated the stability and recyclability of the iron oxide nanocomposites for up to five catalytic cycles, which demonstrates their potential for integration into water treatment methods as a sustainable alternative for the removal of synthetic dyes and other organic pollutants. The results contribute to the development of low-cost, eco-friendly photocatalysts for the efficient removal of complex dye pollutants.

## Supplementary Information

Below is the link to the electronic supplementary material.

Supplementary file 1 (DOCX 3.22 MB)

## Data Availability

All relevant data have been included in the manuscript or as supplementary materials.
