# Supplementary material for: Photocatalytic degradation of single and binary mixture of malachite green and rhodamine B dyes by biochar-capped iron oxide nanocomposites
Source: Environ Sci Pollut Res Int. 2025 Oct 15;32(41):23588–606. doi: 10.1007/s11356-025-37025-8 (PMC12553591; doi:10.1007/s11356-025-37025-8)
Supplement: Supplementary file 1 — (DOCX 3.22 MB) [file 11356_2025_37025_MOESM1_ESM.docx]

**Photocatalytic degradation of single and binary mixture of malachite green and rhodamine B dyes by biochar-capped iron oxide nanocomposites**

Peter A. Ajibade *, Thandi B. Mbuyazi

University of KwaZulu-Natal, School of Chemistry and Physics, Private Bag X01, Scottsville, Pietermaritzburg 3209, South Africa

* Correspondence: [~~ajibadepeters@gmail.com~~](mailto:ajibadepeters@gmail.com) [ajibadep@ukzn.ac.za](mailto:ajibadep@ukzn.ac.za)


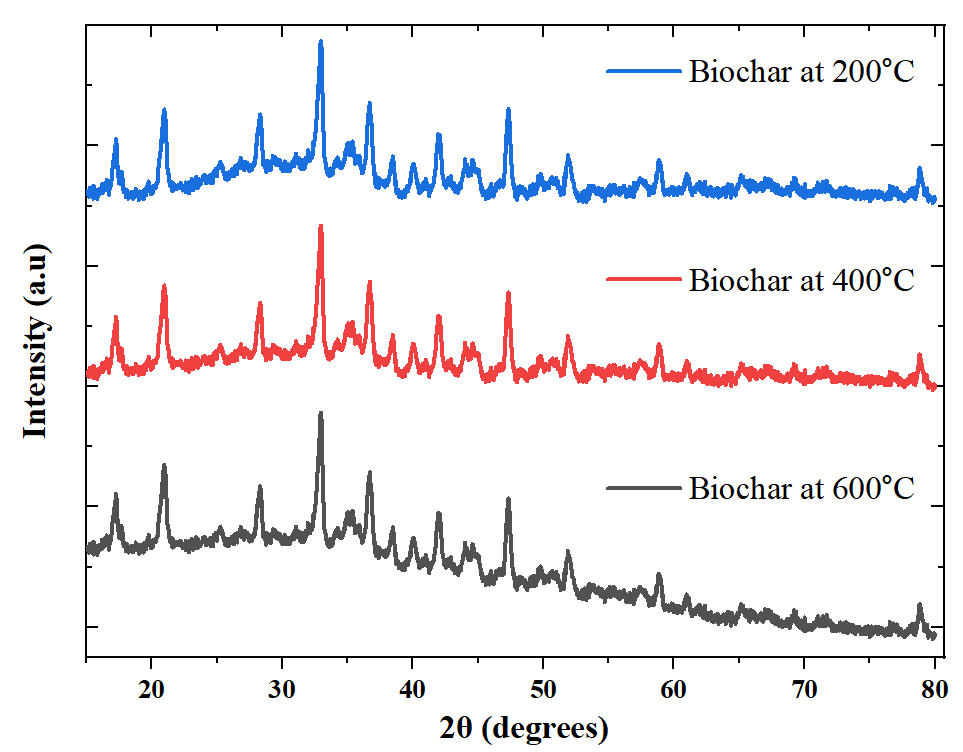


**Fig. S1**. P-XRD of biochar carbonized at different temperatures.


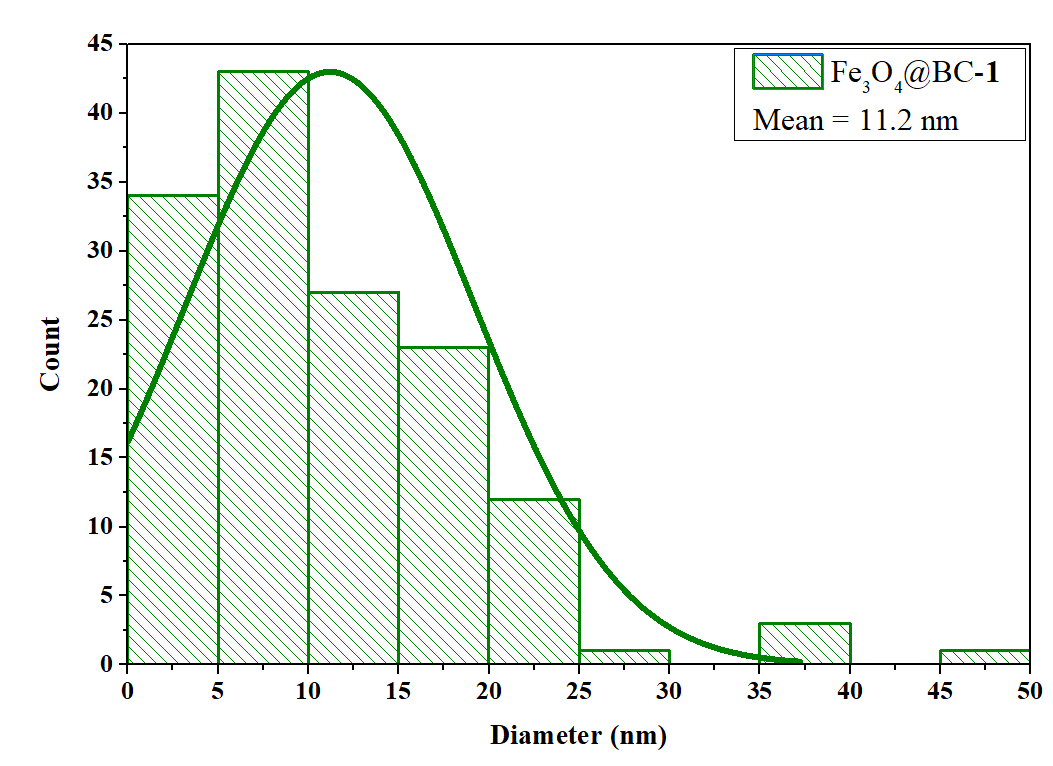


(a)


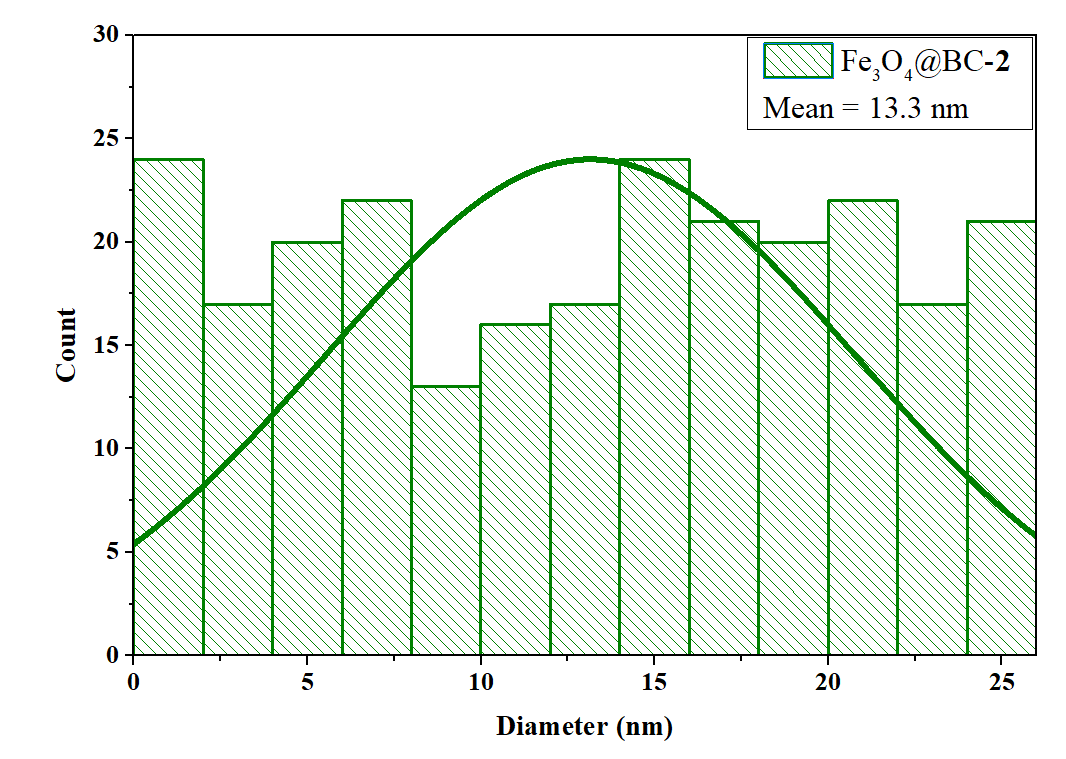


(b)


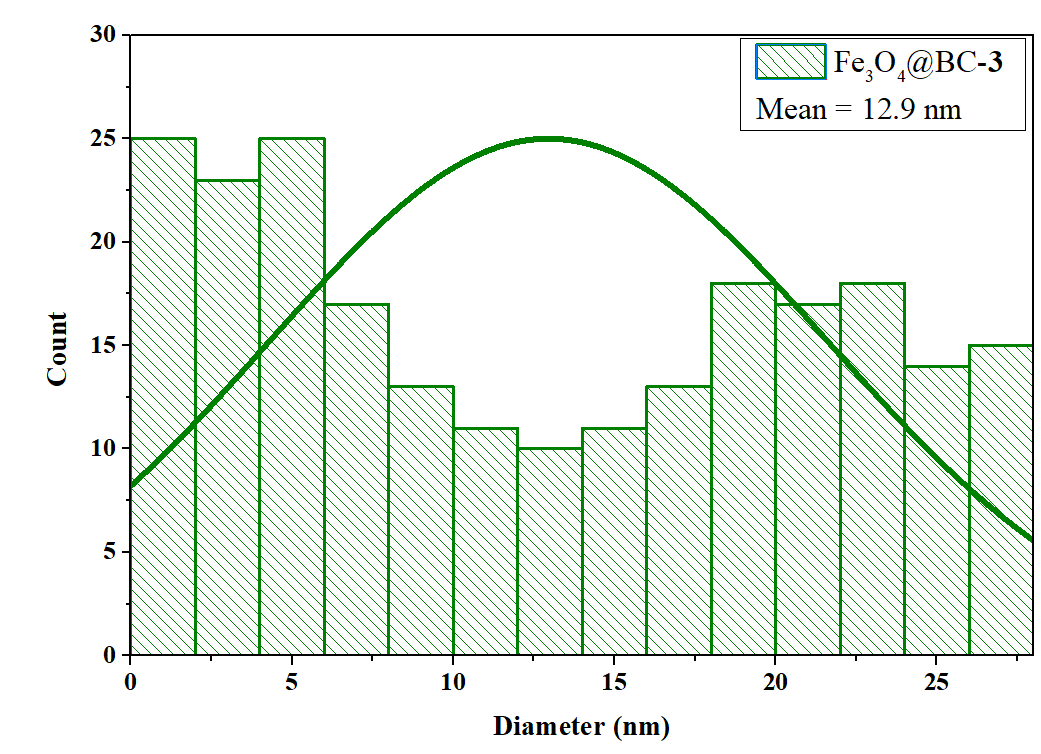


(c)

**Fig. S2**. Size distribution histogram for (a) Fe_3_O_4_@BC–**1**, (b) Fe_3_O_4_@BC–**2** and (b) Fe_3_O_4_@BC–**3** from HRTEM.

**Fig. S3**. SEM and EDX spectra of iron oxide nanocomposites.

| 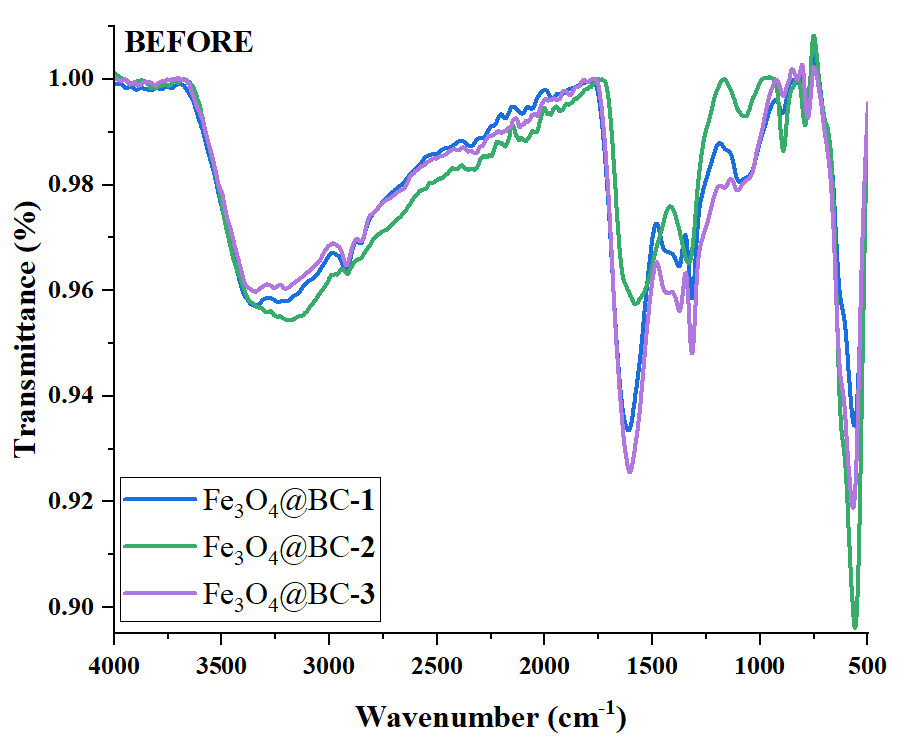  (a) | 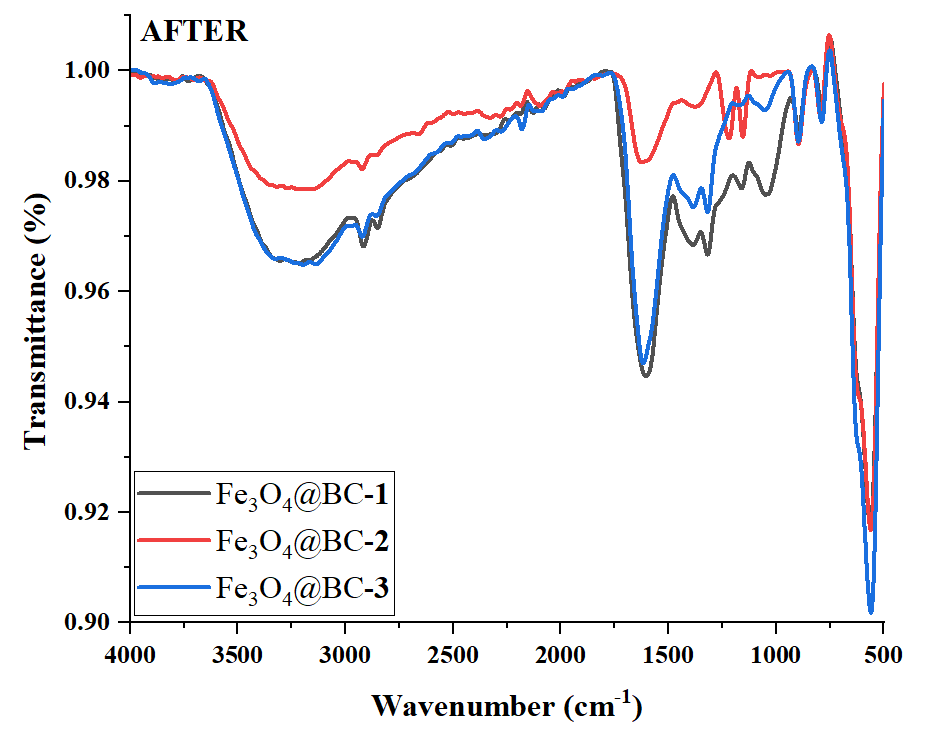  (b) |
| --- | --- |

**Fig. S4**. FTIR spectra of Fe_3_O_4_@BC (a) before and (b) after photocatalytic reaction.

| 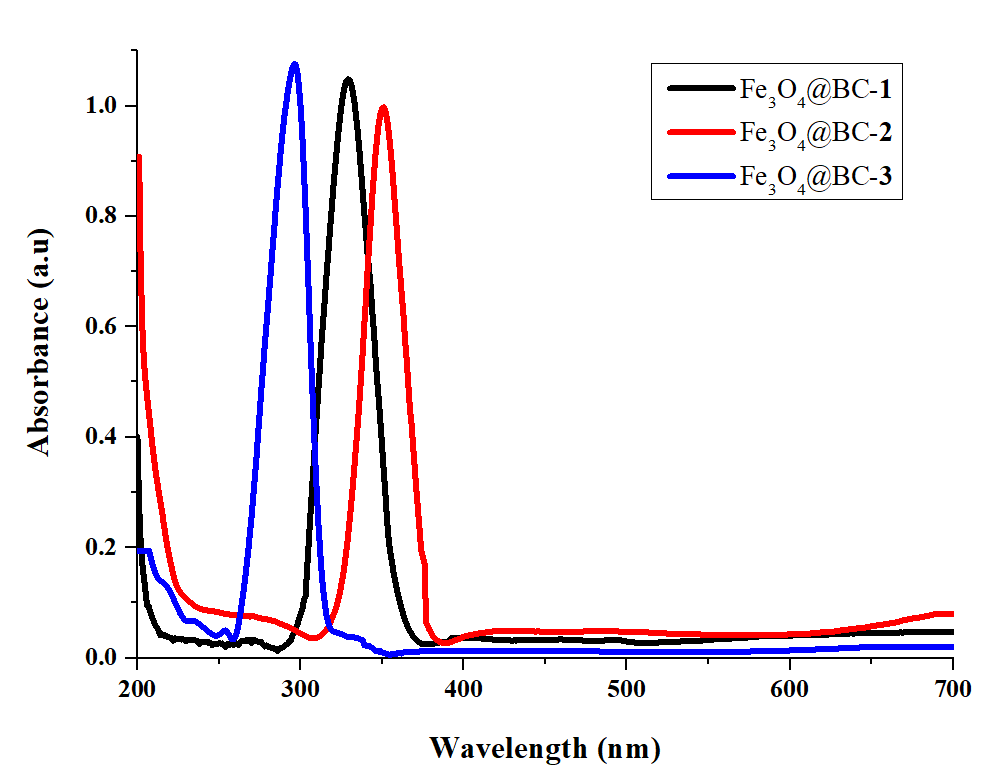  (a) | 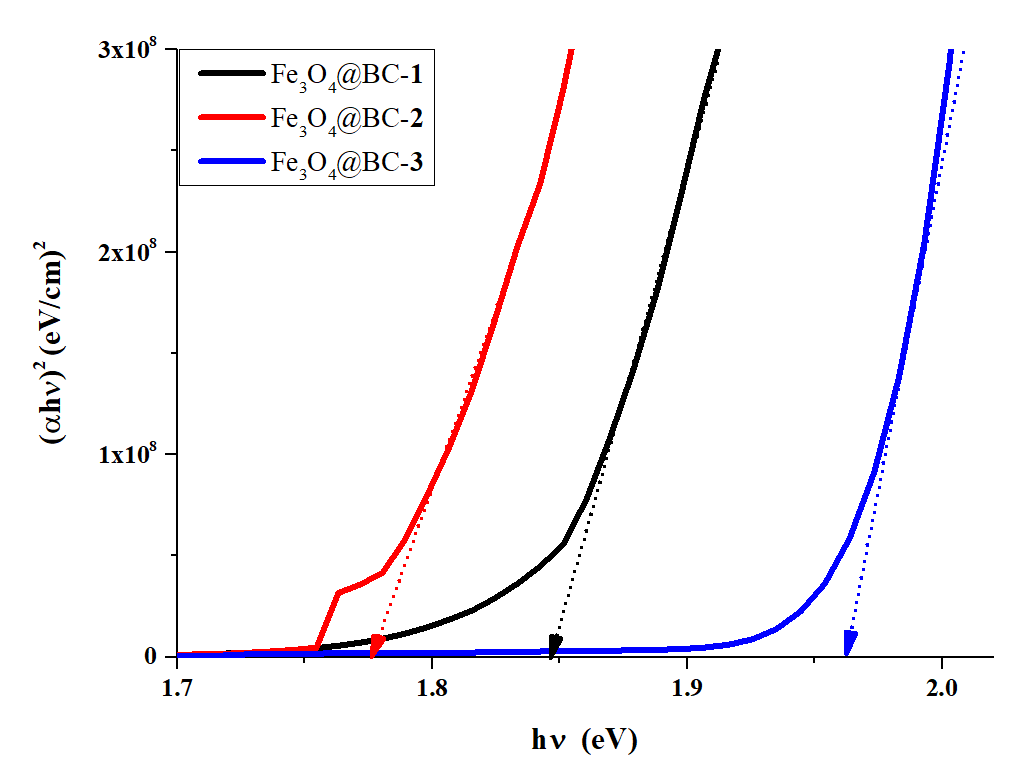  (b) |
| --- | --- |

**Fig. S5.** (a) Absorption spectra and (b) Tauc plots of the biochar-capped iron oxides nanocomposites

**Fig. S6**. Absorption spectra of malachite green degradation over the iron oxide nanocomposites.

**Fig. S7**. Absorption spectra of rhodamine B degradation over the iron oxide nanocomposites.

**Fig. S8**. Absorption spectra of MG-RhB binary dye degradation over the biochar-capped iron oxide nanocomposites.

Table S1. Correlation coefficient and residual sum of squares (RSS) for pseudo-first-order, pseudo-second order, and Langmuir–Hinshelwood models for the degradation of MG, RhB, and dye mixtures by Fe_3_O_4_@BC nanocomposites.

| Dye | catalyst | Pseudo-first order | | Pseudo-second order | | Langmuir–Hinshelwood | |
| --- | --- | --- | --- | --- | --- | --- | --- |
|  |  | R^2^ | RSS | R^2^ | RSS | R^2^ | RSS |
| MG | Fe_3_O_4_@BC-**1** | 0.96850 | 0.02374 | 0.7873 | 24.44268 | 0.96404 | 0.1877 |
|  | Fe_3_O_4_@BC-**2** | 0.95623 | 0.06993 | 0.92072 | 7.97379 | 0.95223 | 0.22301 |
|  | Fe_3_O_4_@BC-**3** | 0.94030 | 0.02335 | 0.97066 | 10.6819 | 0.93226 | 0.5467 |
| RhB | Fe_3_O_4_@BC-**1** | 0.9033 | 0.09892 | 0.96538 | 0.20547 | 0.88404 | 0.09892 |
|  | Fe_3_O_4_@BC-**2** | 0.93002 | 0.07969 | 0.7703 | 1.8319 | 0.91603 | 0.07969 |
|  | Fe_3_O_4_@BC-**3** | 0.93114 | 0.16526 | 0.82056 | 3.5723 | 0.91737 | 0.16526 |
| MG in (MG–RhB) | Fe_3_O_4_@BC-**1** | 0.91913 | 0.43393 | 0.73037 | 372.883 | 0.9136 | 4.44077 |
|  | Fe_3_O_4_@BC-**2** | 0.91972 | 0.31491 | 0.69415 | 7.57326 | 0.913 | 4.34167 |
|  | Fe_3_O_4_@BC-**3** | 0.89624 | 5.16485 | 0.66161 | 2945.15 | 0.89624 | 5.16485 |
| RhB in (MG–RhB) | Fe_3_O_4_@BC-**1** | 0.91465 | 0.2315 | 0.69783 | 2.4737 | 0.91389 | 2.69863 |
|  | Fe_3_O_4_@BC-**2** | 0.91899 | 2.18972 | 0.9165 | 75.614 | 0.91465 | 0.2315 |
|  | Fe_3_O_4_@BC-**3** | 0.94779 | 0.30129 | 0.70467 | 8.8220 | 0.94473 | 0.31159 |

**Fig. S9**. Kinetic plots of single dyes and binary mixture.

(c)

(b)

(a)

**Fig. S10**. Residual sum of squares plots for the pseudo-first order linear fitting of (a) Fe_3_O_4_@BC-**1**, (b) Fe3O4@BC-2, and (c) Fe_3_O_4_@BC-**3** on MG.

(c)

(b)

(a)

**Fig. S11**. Residual sum of squares plots for the pseudo-first order linear fitting of (a) Fe_3_O_4_@BC-**1**, (b) Fe3O4@BC-2, and (c) Fe_3_O_4_@BC-**3** on RhB.

(c)

(b)

(a)

**Fig. S12**. Residual sum of squares plots for the pseudo-first order linear fitting of (a) Fe_3_O_4_@BC-**1**, (b) Fe3O4@BC-2, and (c) Fe_3_O_4_@BC-**3** on MG in (MG-RhB).

(c)

(b)

(a)

**Fig. S13**. Residual sum of squares plots for the pseudo-first order linear fitting of (a) Fe_3_O_4_@BC-**1**, (b) Fe3O4@BC-2, and (c) Fe_3_O_4_@BC-**3** on RhB in (MG-RhB).

**Fig. S14.** Effects of scavengers on single and binary dyes over Fe_3_O_4_@BC nanocomposites.


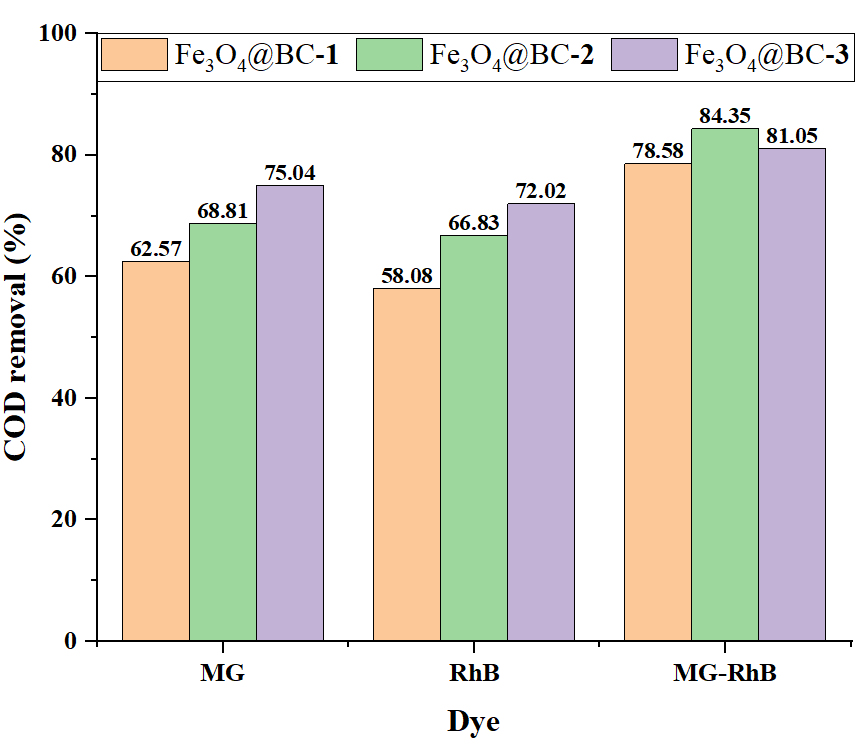


**Fig. S15**. Chemical oxygen demand (COD) removal efficiencies of Fe_3_O_4_@biochar nanocomposites.
